# Dynamic expression of CEACAM7 in precursor lesions of gastric carcinoma and its prognostic value in combination with CEA

**DOI:** 10.1186/1477-7819-9-172

**Published:** 2011-12-23

**Authors:** Jinfeng Zhou, Liyun Zhang, Yong Gu, Kai Li, Yongzhan Nie, Daiming Fan, Yichao Feng

**Affiliations:** 1 State Key Laboratory of Cancer Biology and Xijing Hospital of Digestive Diseases, The Fourth Military Medical University, Number 15, Changle Western Road, Xi'an,710032 China; 2 Department of Digestive Diseases, Affiliated Hospital of Yan'an University, Yan'an, 716000, China

**Keywords:** CEACAM7, gastric carcinoma, precursor lesions, prognosis, CEA

## Abstract

**Background:**

The significance of carcinoembryonic antigen-related cell adhesion molecule 7 (CEACAM7) expression in gastric carcinoma and precancerous lesions and its correlation with CEA expression has rarely been previously investigated.

**Methods:**

CEACAM7 and CEA expression was detected by immunohistochemistry in consecutive sections of 345 subjects with gastric carcinoma and precancerous lesions. Laser confocal analysis was performed to determine CEACAM7 and CEA localization. Correlation between CEACAM7 and CEA expression with clinicopathological parameters was statistically analyzed.

**Results:**

CEACAM7 expression correlated with pathologic grading (P = 0.006), Lauren's classification (P = 0.023), and CEA expression (Spearman R = 0.605, P < 0.001) in gastric carcinoma. CEACAM7 co-localized with CEA predominantly in the cytoplasmic membrane of cancerous cells. CEA expression was correlated with lymph node metastasis (P = 0.031). CEACAM7 and CEA expression increased progressively from precursor lesions to gastric carcinomas. A combination of CEACAM7 and CEA expression was determined to be an independent predictor for patients with gastric carcinoma by multivariate analysis (P = 0.001).

**Conclusions:**

CEACAM7 expression correlates with tumor differentiation and CEA expression in gastric carcinoma. CEACAM7 and CEA expression may synergistically promote gastric carcinogenesis. Combined CEACAM7 and CEA expression analysis can be a useful postoperative predictor for patients with gastric carcinoma.

## Background

In-depth knowledge of the genotypic and phenotypic characteristics of gastric carcinoma and precancerous lesions is extremely important for preventing their development, curtailing their progression to a higher grade tumor once they develop, and providing prognostic information. Gastric carcinomas have generally been divided into intestinal-type and diffuse-type by Lauren [1]. The intestinal-type cancer was thought to display a predominantly intestinal phenotype because it is preceded by a precancerous stage characterized by the sequential steps of atrophic gastritis, intestinal metaplasia, intraepithelial neoplasia (GIN), and intramucosal carcinoma [2,3]. GIN is recognized as an important precancerous lesion, and is categorized as either high- or low-grade according to both the Vienna [4] and WHO [5] classifications. It has been reported that the predominant histologic type may change from the differentiated to the undifferentiated type as the tumors progress [6]. Ohkura, et al also reported that histologic diversity increased as the gastric carcinomas grew or invaded the submucosa [7]. Recent progress in molecular biology has shown that the phenotypic diversity of tumors is associated with a corresponding diversity in gene expression [8-10]. Changes in expression of tumor specific biomarkers in precancerous lesions and various differentiated gastric carcinomas may help us understand the transformation to histological heterogeneity and the underlying molecular mechanism.

The carcinoembryonic antigen (CEA) family of genes has been shown to be expressed in a variety of epithelial derived neoplasms [11], and their functional deregulation has been shown to promote metastases in animal models[12]. One particular CEA gene family member, the CEA cellular adhesion molecule-7 (CEACAM-7), regulates normal cellular differentiation [11]. Deregulation of CEACAM-7 expression has been shown to occur early in colorectal oncogenesis; decreased CEACAM-7 expression was shown in adenomas, hyperplastic polyps, and aberrant crypt foci [11,13]. CEA, another member of the carcinoembryonic antigen family, represents a tumor marker used widely in the management of colorectal cancer [14-16]. Increased CEA levels were the first identified indicator of recurrent disease in 81% [17] and 89% [18] of colorectal cancer patients. Both mesenteric and peripheral levels of CEA were higher in neoplasms with venous involvement, large diameter, and advanced stages of colorectal carcinoma [19]. Increased CEA values have also been reported for other epithelial malignancies, such as those of the breast, lung, and pancreas [20].

To date, little information is available on CEACAM7 expression in gastric nonneoplastic and neoplastic lesions. One study reports that CEACAM7 mRNA levels were up-regulated in cancerous gastric epithelial cells [21]. However, the significance of CEA and CEACAM7 expression in precancerous lesions of gastric carcinoma is poorly understood. Besides, the relationship between CEACAM7 and CEA is unclear, although they belong to the same CEACAM family.

In this study, we investigated the correlation between CEACAM7 and CEA expression, and their relationship to various clinicopathological features in gastric carcinoma, including patient survival. We compared the expression patterns of CEACAM7 and CEA systematically in normal mucosa, chronic atrophic gastritis, gastric intraepithelial neoplasia (GIN), and carcinomas of the stomach with consecutive tissue microarray (TMA) sections. To our knowledge, this is the first immunohistochemical study of CEACAM7 expression in gastric carcinoma and precancerous lesions.

## Methods

### Patients

The study included 345 patients, including 145 patients with gastric carcinoma who underwent primary surgical resection between 2006 and 2009 at the Xijing Hospital of Forth Military Medical University (Xi'an, China), 100 patients with intraepithelial neoplasms (55 low-grade and 45 high-grade), 50 patients with chronic atrophic gastritis, and 50 patients with normal gastric mucosa. The 200 patients with nonneoplastic and precancerous lesions had received a gastric endoscopic check and biopsy between 2008 and 2010 at the Xijing Hospital. All diagnoses, including differentiation status, were made based on the Pathology and Genetics Tumors of Digestive System by three pathologists [22]. GIN is categorized as either high- or low-grade according to both the Vienna [4] classifications. Follow-up was performed on the 145 patients with gastric carcinoma for survival analysis. These patients underwent subtotal gastrectomy with D2 lymph node dissection at Xijing Hospital from 2006 to 2009. The mean age was 56.4 years (range, 32-74 years). All patients received postoperative chemotherapy using a fluorouracil-based regimen. No patients received preoperative chemotherapy or underwent radiotherapy. Follow-up was performed on patients from the date of surgery until either the date of death or March 30, 2011, resulting in follow-up periods ranging from 5 to 67 months (mean, 31 months). Those cases lost to follow-up or those who died of a cause other than gastric cancer were regarded as censored data for the analysis of survival rates. In all cases, informed consent was obtained for the use of resected tumor specimens. This study was approved by the Moral and Ethical Committee of the Xijing Hospital of Forth Military Medical University.

### Tissue Microarrays

Tissues of gastric carcinoma or precancerous lesions were made into tissue micro-arrays by Tissue Microarrayer (Beecher Instruments, Silver Spring, USA ™), according to the technique of Kononen et al [23]. Briefly, core tissue biopsies (2 mm in diameter) were taken from individual paraffin-embedded tissues (donor blocks) and arranged in a new recipient paraffin block (tissue array block) using a trephine apparatus. The staining results of the different areas in these tissue array blocks showed excellent agreement. One core was chosen from each case for analysis. We defined an adequate case as a tumor that occupied 10% of the core area.

### Primary antibodies for immunohistochemistry

Mouse anti-human CEACAM7 antibody [BAC2] (ab26281) and Rabbit Anti-human CEA antibody (ab-15157) was all purchased from Abcam plc. (Cambridge, UK).

### Immunohistochemistry

Immunohistochemistry was performed on 4-lm-thick, routinely processed, paraffin sections in series. Briefly, after baking on a panel at 60°C for an hour, the paraffin embedded, formalin fixed tissue samples were deparaffinized with xylene and rehydrated through gradient ethanol immersion. Endogenous peroxidase activity was quenched by 3% (vol/vol) hydrogen peroxide in methanol for 10 minutes, followed by three 3-minute washes with phosphate buffered saline (PBS). This was followed by a step of antigen retrieval. The slides were immersed in 0.01 mol/l citrate buffer solution (pH 6.0) and placed in a microwave oven for 30 min. After a wash in 0.01 mol/l of phosphate-buffered saline (PBS, pH 7.4), The sections were then blocked with 10% (vol/vol) normal goat serum in PBS for 30 minutes followed by incubation in a moist chamber at 4 overnight with the primary antibody to CEACAM7 (1:100, ab26281, Abcam) or CEA 1:10, ab-15157, Abcam) diluted in PBS containing 1% (wt/vol) bovine serum albumin (BSA). Negative controls were performed by replacing the primary antibody with pre-immune mouse serum. After three 3-minute washes with PBS, the sections were treated with second anti-mouse antibody (PV-6002, Santa Cruz) for 30 minutes at room temperature followed by additional three 3-minute washes with PBS. Reaction product was visualized with diaminobenzidine (DAB, ZLI-9032, ZSGB. Beijing, China) at room temperature for 2 min. After being counterstained with Harris hematoxylin (ZLI-9039, ZSGB. Beijing, China) for 3 min, and rinsed with tap water, the sections were immediately dehydrated by sequential immersion in gradient ethanol and xylene, and mounted with pernount and cover slips. Images were obtained under a light microscope (Olympus BX51, Olympus, Japan) equipped with a DP70 digital camera.

### Evaluation of Staining

For evaluation of cell staining, sections were examined by two independent pathologists without prior knowledge of the clinicopathological status of the specimens. Expression of CEACAM7 and CEA was evaluated according to the ratio of positive cells per specimen (R) and staining intensity (I). The ratio of positive cells per specimen was scored 0 for staining of < 1%, 1 for staining of 2 to 25%, 2 for staining of 26 to 50%, 3 for staining of 51 to 75%, and 4 for staining > 75% of the cells examined. Intensity was graded as following: 0, no signal; 1, weak; 2, moderate; and 3, strong staining. A total score (R × I) of 0 to 12 was finally calculated and graded as negative (-score: 0-2) and positive (+, 3-12).

### Immunofluorescence and laser confocal scanning

After baking on a panel at 60°C for an hour, the sections were deparaffinized with xylene and rehydrated through gradient ethanol immersion. This was followed by a step of antigen retrieval. The slides were immersed in 0.01 mol/l citrate buffer solution (pH 6.0) and placed in a microwave oven for 30 min. After a wash in 0.01 mol/l of phosphate-buffered saline (PBS, pH 7.4), samples were incubated with blocking buffer (5% normal goat serum, 1% bovine serum albumin and 0.1% triton X-100) for one hour at room temperature. Samples were then incubated with primary antibodies diluted in the blocking buffer at 4°C overnight, followed by 3 washes in PBS for 10 minutes each. Finally, samples were stained with secondary antibodies diluted in blocking buffer for 30 minutes followed by 3 washes in PBS. Coverslips were mounted on slides with Prolong Antifade (Invitrogen). Antibodies used for immunostaining include mouse anti-CEACAM7 (1:100, ab26281, Abcam), rabbit anti-CEA (1:10, ab-15157, Abcam), secondary antibodies used were goat anti-mouse IgG Alexa 549 (1:300, Invitrogen), goat anti-rabbit IgG Alexa 488 (1:300, Invitrogen), DAPI (1:1000, Molecular Probes). Adjacent normal tissues were used as negative controls (20 mm away from the cancer tissue). Immunostained tissues were visualized and images were captured by using FlUOVIEW (FVLIOi, OLYMPUS.

### Statistical Analysis

All statistical analyses were performed using IBM SPSS 19.0 software. Measurement data were analyzed using Student's t or one-way ANOVA test, while categorical data were studied using theχ2 or nonparametric test. Survival curves were estimated using the Kaplan-Meier method, and the log-rank test was used to calculate differences between the curves. Multivariate analysis using the Cox proportional hazards regression model was performed to assess the prognostic values of protein expression. Correlation coefficient between expression of CEACAM7 and CEA was estimated using the Spearman correlation method. Statistical significance was set at P < 0.05.

## Results

### Co-expression of CEACAM7 and CEA in gastric carcinoma

**Figure 1:**
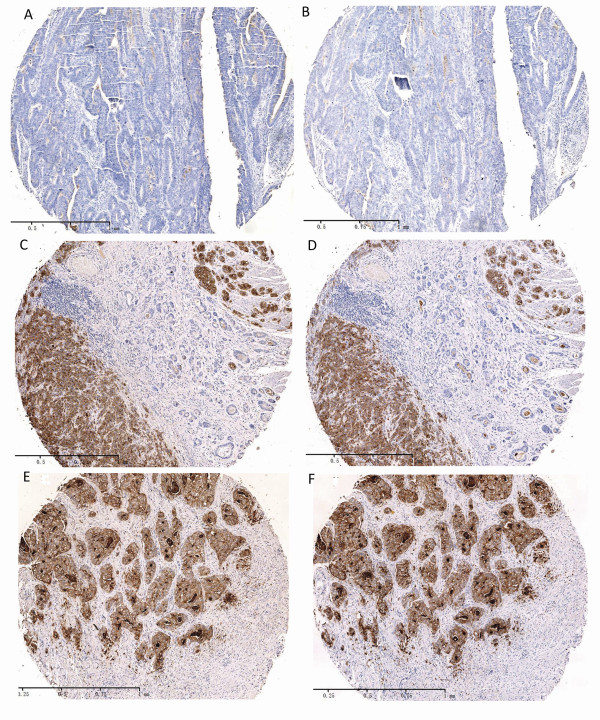
**Co-expression of CEACAM7 and CEA in gastric carcinomas**. A&B, Negative staining for CEACAM7 (A) and CEA (D) in a well differentiated gastric carcinoma. C&D, CEACAM7 (C) and CEA (D) staining was negative in the well differentiated area (cancer cells are ranged in gland-like form) and positive in the poorly differentiated area (brown color) of a gastric carcinoma. E&F, Positive staining for CEACAM7 (E) and CEA (F) in a poorly differentiated gastric carcinoma. A&B, C&D, and E&F are adjacent serial sections, respectively.(DAB as chromogen, counterstained with Harris hematoxylin, 50X).

**Figure 2:**
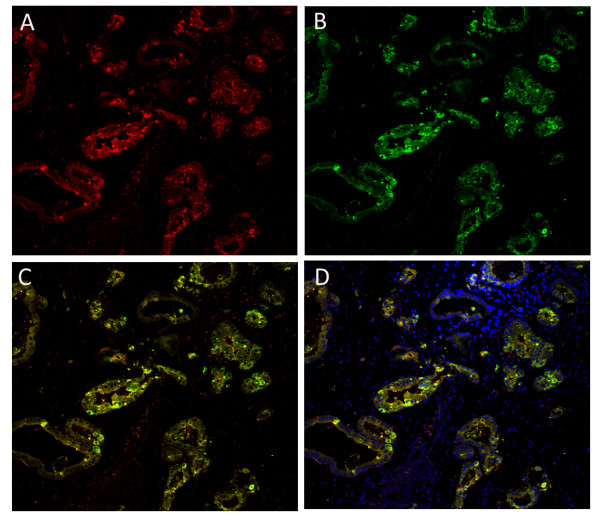
**Co-localization of CEACAM7 and CEA in the tissue of gastric carcinoma (Laser confocal microscopy analysis in a representative section)**. Positive expression for CEACAM7 (A, red) or CEA (B, green) in the membrane and cytoplasm of cancerous cells (red). C, Merged image without nuclear staining; co-expression of CEACAM7 and CEA is observed (yellow). D, Merged image with nucleus stained by DAPI (blue). (200X).

Immunohistochemistry was performed on consecutive sections of 145 gastric carcinomas using either anti-CEACAM7 or anti-CEA antibody (Figure 1). Immunohistochemical analysis shows that 100 of 145 (69.0%) samples were categorized as CEACAM7-negative (Figure 1A), and 45 of 145 (31.0%) samples were categorized as CEACAM7-positive (Figure 1C, 1E). CEACAM7 expression was concordant with CEA expression in 80.0% (116 of 145) of gastric carcinoma cases (Spearman R = 0.605, P < 0.001). CEACAM7 and CEA double-positivity was observed in 40 cases, double-negativity was observed in 76 cases, and 29 cases were found to be either CEACAM7-positive or CEA-positive only. The expression correlation coefficient was estimated using the Spearman Rank Correlation method. The Spearman R value was 0.605 (P < 0.001), indicating a close correlation between CEACAM7 and CEA expression in gastric carcinomas. As it is inconvenient and unnecessary to check the localization of the two antigens by immunofluorescence in all the 40 carcinoma tissues that are positive for both CEACAM7 and CEA, we investigated their localization in seven tissue sections that were randomly selected from the 40 carcinoma tissues sections. Laser confocal microscopy analysis showed that they co-localized in the membrane and cytoplasm of cancerous cells (Figure 2A,B,C,D).

### Correlation of CEACAM7 and CEA expression with clinicopathological features of gastric carcinoma

Relationship between CEACAM7 and CEA expression and various clinicopathological features of gastric carcinomas is summarized in Table 1. CEACAM7 was more frequently expressed in poorly differentiated tumors than in well and moderately differentiated gastric carcinomas (41.3% vs. 20.0%, P = 0.006). CEACAM7 positivity was significantly higher in diffuse-type gastric carcinomas than in intestinal-type gastric carcinomas, (39.7% vs. 22.2%, P = 0.023). CEA expression significantly correlated with lymph node metastasis (P = 0.031).

**Table 1:** Correlation of CEACAM 7 and CEA expression with clinicopathological characteristics of gastric carcinoma.

Variables	CEACAM 7		P value	CEA		P value
				
	Negative (n = 100)	Positive (n = 45)		Negative (n = 81)	Positive (n = 64)	
Gender						
Male	74(67.3%)	36(32.7%)	0.437	58(52.7%)	52(47.3%)	0.178
Female	26(74.3%)	9 (25.7%)		23(65.7%)	12(34.3%)	
Age (Years)						
(means ± SD)	59.8 ± 10.4	58.1 ± 13.3	0.608	60.3 ± 9.4	58.9 ± 12.9	0.512
Location of tumor						
Upper	12(60.0%)	8 (40.0%)	0.601	9 (45.0%)	11(55.0%)	0.544
Middle	49(69.0%)	22(31.0%)		40(56.3%)	31(43.7%)	
Lower	39(72.2%)	15(27.8%)		32(59.3%)	22(40.7%)	
Histology †						
Well and Moderate	56(80.0%)	14(20.0%)	0.006	43(61.4%)	27(38.6%)	0.741
Undifferentiated	44(58.7%)	31(41.3%)		38(50.7%)	37(49.3%)	
Lauren's classification						
Intestinal	56(77.8%)	16(22.2%)	0.023	39(54.2%)	33(45.8%)	0.683
Diffuse	44(60.3%)	29(39.7%)		42(57.5%)	31(42.5%)	
Depth of tumor						
T1-T2	28(75.7%)	9 (24.3%)	0.307	20(54.1%)	17(45.9%)	0.797
T3-T4	72(66.7%)	36(33.3%)		61(56.5%)	47(43.5%)	
Node metastasis						
Negative	59(72.8%)	22(27.2%)	0.257	37(67.3%)	18(32.7%)	0.031
Positive	41(64.1%)	23(35.9%)		44(48.9%)	46(51.1%)	
Lymphatic invasion						
Negative	44(75.9%)	14(24.1%)	0.143	35(60.3%)	23(39.7%)	0.375
Positive	56(64.4%)	31(35.6%)		46(52.9%)	41(47.1%)	
Venous invasion						
Negative	52(70.3%)	22(29.7%)	0.729	43(58.1%)	31(41.9%)	0.578
Positive	48(67.6%)	23(32.4%)		38(53.5%)	33(46.5%)	
Tumor Stage						
I- II	40(62.5%)	24(37.5%)	0.135	34(53.1%)	30(46.9%)	0.555
III- IV	60(74.1%)	21(25.9%)		47(58.0%)	34(42.0%)	

NOTE: Pearson's X2 test was done to get P value except that Ages was compared by Student-T test. †Well = well differentiated carcinoma; moderately = moderately differentiated carcinoma; undifferentiated = poorly differentiated carcinoma, signet ring cell carcinoma, or mucinous carcinoma.

### Dynamic expression of CEACAM7 and CEA in normal mucosa and precancerous lesions

**Figure 3:**
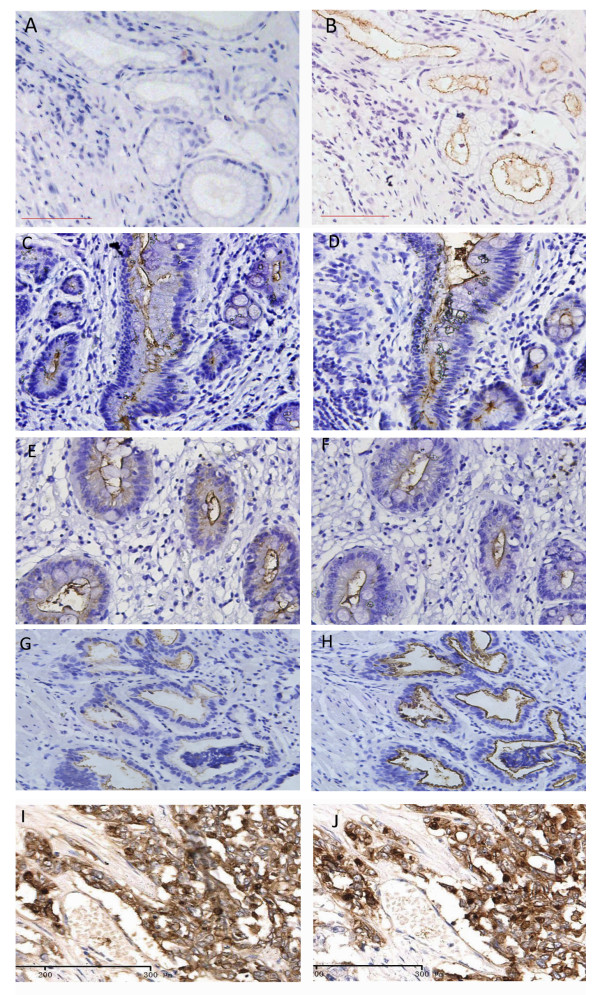
**Different expression patterns of CEACAM7 and CEA in gastric non-neoplastic lesions and gastric carcinomas**. Consecutive sections were used for analysis. No immunoreaction for CEACAM7 was detected in normal mucosa (A). CEACAM7 was localized on the apical surface of chronic atrophic gastritis (C), low grade GIN (E) and high grade GIN (G). CEA was localized on the apical luminal surface of normal mucosa (B), chronic atrophic gastritis (D), low-grade GIN(F), and high-grade GIN (H). Both CEACAM7 (I) and CEA (J) was localized on the whole surface of gastric cancerous cells. (DAB as chromogen, counterstained with Harris hematoxylin, 200X).

The results of CEACAM7 and CEA expression in normal mucosa and precancerous lesions were summarized in Table 2. Positivity for CEACAM7 expression was found in chronic atrophic gastritis (12%; 6/50), low-grade GIN (29.1%; 16/55), and high-grade GIN (28.9%; 13/45). CEACAM7 expression was not found in normal mucosa (0/50). Positivity for CEACAM7 expression in low grade GINs was significantly higher than for chronic atrophic gastritis (P = 0.032), and CEACAM7 was more frequently expressed in atrophic chronic gastritis than in normal gastric mucosa (P = 0.012), but there was no significant difference in CEACAM7 expression between low-grade GIN and high-grade GIN (P = 0.982). CEA positivity was found in normal mucosa (6%; 3/50), chronic atrophic gastritis (8%; 4/50), low-grade GIN (57.1%; 13/55), and high-grade GIN (60%; 20/45). The CEA positivity for low-grade GIN was significantly higher than for chronic atrophic gastritis (P = 0.030), but was lower than that for high grade GIN (P = 0.028). There was no significant difference in CEA positivity between normal mucosa and chronic atrophic gastritis (P = 0.500). CEACAM7 expression was localized to the luminal surface of chronic atrophic gastritis and GINs (Figure 3C, E, and 3G), no immunoreaction was detected in normal mucosa (Figure 3A). CEA was localized on the luminal surface of normal mucosa, chronic atrophic gastritis, low-grade GIN, and high-grade GIN (Figure 3B, D, F, and 3H), whereas both CEA and CEACAM7 expression was localized to the entire surface of gastric cancerous cells (Figure 3I and 3J)

**Table 2:** Relation between CEACAM7 and CEA expression with different gastric tissues

Tissues	N	CEACAM7			CEA		
					
		Negative	Positive	P value*	Negative	Positive	P value*
Normal mucosa	50	50 (100%)	0(0%)	0.012^1^	47(94%)	3(6%)	NS^4^
Chronic atrophic gastritis	50	44(88.0%)	6(12.0%)	0.032^2^	46(92%)	4(8%)	0.030^5^
Low grade GIN	55	39(70.9%)	16(29.1%)	NS^3^	42(76.4%)	13(23.6%)	0.028^6^
High grade GIN	45	32 (71.1%)	13(28.9%)		25(55.6%)	20(44.4%)	

*χ2 test. N-number of cases, GIN-gastric intraepithelial neoplasia. NS-no significance (P > 0.05). ^1 ^Compared with normal mucosa, CEACAM7 positivity was significantly higher in chronic atrophic gastritis(P = 0.012); ^2 ^compared with chronic atrophic gastritis, CEACAM7 was more frequently expressed in Low grade GIN(P = 0.032); ^3^There was no significant difference of CEACAM7 expression between Low grade GIN and High grade GIN(P = 0.982); ^4^There was no significant difference of CEA expression between normal mucosa and chronic atrophic gastritis(P = 0.500); ^5^Compared with chronic atrophic gastritis, CEA was more frequently expressed in Low-grade GIN(P = 0.030); ^6 ^Compared with Low grade GIN, CEA was more frequently expressed in High grade GIN (P = 0.028).

### Survival Analysis

**Figure 4:**
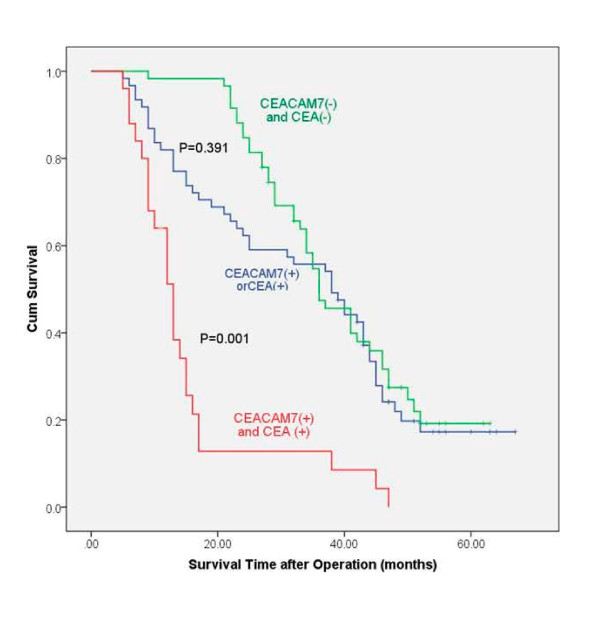
**Kaplan-Meier curves for postoperative survival**. The median survival time of patients with CEACAM7 and CEA double-positivity was shorter than that of patients with double-negativity (log-rank test: P = 0.001). But, there was no significant difference between the median survival time of patients with single-positivity and double-negativity (P = 0.391).

One-year and three-year survival rates were 92.0% and 54.1%, respectively, for CEACAM7 and CEA double-negative patients, 98.3% and 45.1%, respectively, for CEACAM 7 and CEA single-positive (either CEACAM7 positive or CEA positive) patients, and 64.0% and 8.5% for CEACAM7 and CEA double-positive patients. Significant difference was observed between the survival rate of double-negative and double-positive patients (Figure 4, P = 0.001). However, no significant difference was found between double-negative and single-positive patients (Figure 4, P = 0.391). Multivariate analysis demonstrated that double-positivity for CEACAM7 and CEA expression (P = 0.004) and tumor stage (P = 0.001) were factors independently associated with poorer patient prognosis (Table 3).

**Table 3:** Multivariate analysis based on Cox's proportional hazards model

Variable	Hazard Ratio (95% Confidence Interval)	P-value
Tumor Stage III, IV vs I, II	2.240 (1.501-3.343)	0.001
CEACAM7(+)CEA(+) vs CEACAM7(-)CEA(-)	1.545 (1.153-2.069)	0.004

## Discussion

CEA is one of the most useful tumor markers for carcinoma[24], and its expression was found to be correlated with clinicopathological features, such as venous involvement, greater diameter, and advanced stages of colorectal carcinomas[25,26]. In colon cancer, CEA is upregulated, but CEACAM7 was reported to be downregulated[11], it is interesting that they were both upregulated in gastric cancer, and co-expressed in most of the tissues. suggesting that CEACAM7 may play different roles in different cancers. As CEACAM7 expression was found to be closely correlated with CEA expression in gastric carcinoma in this study, We checked its correlation with various clinicopathological features, and found that CEACAM7 expression was more frequent in poorly differentiated gastric carcinomas than in well and moderately differentiated carcinomas (P = 0.006), and was also correlated with Lauren's classification (P = 0.023). It has been reported that the predominant histologic type may change from the differentiated to the undifferentiated type as the tumors progress [6], Ohkura, et al[17] also reported that histologic diversity increases as gastric carcinomas grow or invade the submucosa. Thus, we hypothesized that CEACAM7 might promote aggressiveness and progression of gastric carcinoma via regulating tumor cell differentiation, and this needs further investigation.

As intestinal-type cancer is thought to be preceded by a precancerous stage characterized by the sequential steps of atrophic gastritis, intestinal metaplasia, GINs, and intramucosal carcinoma, it would be of great significance to clarify the dynamic expression of CEACAM7 and CEA in normal gastric mucosa, chronic atrophic gastritis, GINs, and gastric carcinoma. We found that, compared with gastric normal mucosa, CEACAM7 expression was significantly increased in chronic atrophic gastritis and all other lesions, and CEA expression was significantly increased in GINs and gastric carcinoma. These results indicate that CEACAM7 may play a role in gastric carcinogenesis from an early stage, specifically the chronic atrophic gastritis stage. Furthermore, CEA may cooperate with CEACAM7 at the GINs stage. Consistent with our results, CEA transgenic mice showed massively enlarged colons comprising a continuous mosaic of severe hyperplasia, dysplasia, and serrated adenomatous morphology, suggesting that up-regulation of CEA could be an instrumental step in human cancer progression [27]. It appears that up-regulation of CEACAM7 or CEA expression may be an early molecular event in tumorigenesis, and both proteins could be used as screening biomarkers in precancerous lesions to identify patients with a high risk of malignant conversion. In contrast, decreased expression of CEACAM-7 has been shown to occur early in colorectal oncogenesis, with decreased expression in adenomas, hyperplastic polyps, and even aberrant crypt foci.11,13 The contrasting expression of CEACAM7 in gastric and colorectal carcinogenesis implies that its function is context dependent.

Patients with advanced cancer often want to know how long they have left to live [28], but clinicians are not confident at estimating prognosis [29]. Therefore, biomarkers for prognosis are a potential tool to help clinicians with patient care. During the past decade, a large number of proteins that are putatively important in carcinogenesis and cancer biology have been studied for their prognostic value in gastric cancer, but none have been proven to be sufficiently useful in clinical prediction. It is highly unlikely that a single protein marker will provide the sensitivity and specificity required for prognosis. Thus, emphasis has shifted to the discovery of combinations of biomarkers directly related to disease processes [30]. In this study, only advanced tumor stage and CEACAM7 and CEA double-positivity were identified using the Cox proportional hazards model as independent prognostic predictors for the survival of patients with resectable gastric carcinoma, independent of age, gender, differentiation, pathologic TNM stage, lymph node metastasis, lymphatic, and venous invasion of the patients.

## Conclusions

To our knowledge, this is the first comparative immunohistochemical study of CEACAM7 and CEA expression in gastric carcinoma and precancerous lesions. The two proteins were co-expressed and co-localized in gastric carcinoma. CEACAM7 expression was found to be significantly correlated with the differentiation of gastric carcinoma. The expression of CEACAM7 and CEA was found to increase gradually during the development of gastric carcinoma. CEACAM7 and CEA double-positivity may be a useful postoperative prognostic predictor for patients with gastric carcinoma.

## Competing interests

The authors declare that they have no competing interests.

## Authors' contributions

JZ and LZ supervised research project, participated in the data collection, and drafted the manuscript. KL and YG participated in the data collection, supervised ICH. YN and DF acted as corresponding authors and did the revisions. All authors read and approved the final manuscript. JZ and LZ contribute equally to this work.
